# Reliability and validity of Thai versions of the MOS-HIV and SF-12 quality of life questionnaires in people living with HIV/AIDS

**DOI:** 10.1186/1477-7525-9-15

**Published:** 2011-03-15

**Authors:** Suwat Chariyalertsak, Tanyaporn Wansom, Surinda Kawichai, Cholthicha Ruangyuttikarna, Verne F Kemerer, Albert W Wu

**Affiliations:** 1 Research Institute for Health Sciences, Chiang Mai University PO Box 80 CMU, Chiang Mai 50202 Thailand; 2 Department of Medicine Johns Hopkins Bayview Medical Center 4940 Eastern Avenue, B1 N 1st Floor, Baltimore, MD 21224 USA; 3 UN Trust Fund to End Violence Against Women 2345 Crystal Drive, Suite 301, Arlington, VA 22202 USA; 4 Johns Hopkins Bloomberg School of Public Health 624 N. Broadway, Rm. 653, Baltimore, MD, 21205 USA

## Abstract

**Background/Aim:**

As Thai people living with HIV/AIDS gain increasing access to antiretroviral (ARV) therapy, it is important to evaluate the impact this has not only on clinical outcomes, but also on patients' functional status and well-being. In this study, we translated, culturally adapted and tested the reliability and validity of two widely-used health-related quality of life questionnaires - the MOS-HIV Health Survey and the SF-12 - in people living with HIV/AIDS in Northern Thailand. Methods: Questionnaires were administered to 100 patients at community hospital outpatient ARV clinics in northern Thailand. Reliability was estimated using Cronbach's alpha, while evidence for validity was tested using known-groups comparison based on CD4 group, symptom distress score, bed days and days of reduced activity in the past three months.

**Results:**

Patients' median age was 36, with 58% female, 58% working as laborers, and 60% completing at least primary education. Median CD4 count was 218 cells/mm^3^. There were no missing data. For the MOS-HIV and SF-12, mean physical summary scores were 53.1 and 49.0 respectively; mean mental summary scores were 53.4 and 45.6, respectively. Internal consistency coefficients were >0.7 for all but one scale, the PF scale (0.67). As hypothesized, scores were slightly to moderately correlated with CD4 count, symptom score, number of days in bed or with reduced activity. Correlations were higher with physical health scores than with mental health scales. The MOS-HIV discriminated clinical known groups slightly better than the SF-12.

**Conclusion:**

Both the MOS-HIV and the shorter SF-12 were successfully adapted for people with HIV/AIDS in Northern Thailand, and showed encouraging evidence for reliability and validity. These patient reported questionnaires could be valuable tools in evaluating therapeutic interventions and other innovations in health and social services, and to estimate health needs and population disability related to HIV.

## Introduction

With the introduction of generic antiretroviral therapy into the world market, the number of people accessing antiretroviral therapy globally continues to rise. Access to appropriate antiretroviral therapy offers hope for decreased morbidity and mortality to those living with HIV/AIDS. However, leaders of multi-national trials have cautioned against defining success by using only clinical endpoints such as lab results (CD4+ T lymphocyte count, HIV viral load) as these measures cannot capture the complexity of a patient's experience on antiretroviral treatment [1,2]. To achieve comprehensive evaluation, health-related quality of life (HRQOL) measures have become increasingly important to help assess the impact treatment has on patients' lives.

Thailand has often been held up as a model for effective HIV control and prevention in low and middle income countries. Thailand has a strong health systems infrastructure and is also a regional hub for pharmaceutical production [3]. Because of this, Thailand was able to guarantee universal access to antiretroviral treatment through the National Access to Antiretroviral for People Living with HIV/AIDS (NAPHA) program, passed in 2001 [4]. With the scale-up of antiretroviral (ARV) treatment, increasing numbers of people have been able to access antiretroviral treatment. Furthermore, patients have been able to remain on treatment longer than they might have otherwise because of the ability of physicians to switch regimens under the NAPHA plan. According to the Global Fund, in 2008 more than 140,000 people were receiving on ARV in Thailand [5]; however, HRQOL has rarely been assessed in the Thai setting outside of clinical trials. To accomplish this, it is necessary to identify HRQOL measures that are reliable, culturally appropriate and conceptually equivalent to existing measures. This will allow interpretation of the results from Thai studies, as well as comparison to other studies both within country and globally.

A number of health-related quality of life measures have been developed for HIV-infected patients and include assessment of various domains, including functional status and psychological well-being [6,7]. The purpose of this study was to translate, culturally adapt, and test the reliability and validity of two widely-used HRQOL measures, the Medical Outcomes Study-HIV (MOS-HIV) and Short Form Health Survey-12 (SF-12), among Thai people living with HIV/AIDS currently or previously on HAART. A third tool, the AIDS Clinical Trials Group (ACTG) symptom distress module (SDM) [8] was also translated and culturally adapted into Thai and pilot tested among the same group of patients. In addition to estimating the reliability of the instruments, we also compared their ability to discriminate known groups based on CD4 count, symptom distress score, number of days spent in bed and number of days where activity had to be reduced due to health status.

## Methods

The study design included two phases: translation/cultural adaptation and pilot testing. During the first stage, the MOS-HIV, SF-12, and ACTG SDM were translated and culturally adapted from the original US English into Thai. In the second stage, these questionnaires were used in a cross-sectional survey of HIV+ patients on HAART in two community-based district hospitals in Northern Thailand.

### Questionnaires

The MOS-HIV and SF-12 were culturally adapted and translated using the linguistic validation method developed by the MAPI Research Institute [9]. This method aims to achieve conceptual equivalence rather than literal translation and has been used successfully to translate many patient-reported outcome instruments into a variety of languages.

The MOS-HIV is a 35 item questionnaire that was specifically designed to measure QOL in patients with HIV. The MOS-HIV has two summary scores for mental and physical health, and 10 subscales which include the following dimensions: general health perceptions, pain, physical functioning, role functioning, social functioning, cognitive functioning, mental health, vitality (energy/fatigue), health distress and quality of life [10].

The SF-12 is a brief, generic 12-item questionnaire. It assesses eight dimensions of HRQOL: physical functioning, role limitations due to physical health, role limitations due to emotional health, social functioning, bodily pain, general health perceptions, vitality, and mental health(4). Two summary scores are generated - a physical component score (PCS) and a mental component score (MCS)[10].

The AIDS Clinical Trials Group (ACTG) Symptom Distress Module (SDM) is a patient-reported index that asks the patient to state whether he/she has a symptom and then to quantify how much that symptom bothers him or her [11]. Responses are quantified on a Likert-type scale with response items ranging from 0 (I do not have this symptom) to 4 (I have this symptom, and it is a big problem for me). The 22 items within the symptom score address an array of issues, including sleep, appetite, depression, weight, and sexual dysfunction that are not captured in many traditional QOL measures. Higher symptom scores indicate both an increased incidence in symptoms as well as a larger negative effect these symptoms have on the patient's QOL.

### MAPI method of translation/cultural adaptation

The MAPI method of linguistic validation comprises three major steps: 1) forward translation from the source, or original, language into the target language (in this case, Thai), 2) backward translation, and 3) patient testing. This method is similar to the one used by the International Quality of Life Assessment (IQOLA) project, which translated and culturally adapted the SF-36, SF-12, and SF-8 into more than 40 languages [9,11,12].

In the first step, two independent translators produce their own forward translated versions of the instrument. All parts of the questionnaire are translated, including instructions for completing the questionnaire, original questions, and response items. Once the two independent versions are completed, each version is back-translated into the source language by two separate translators. Finally, a harmonized version is formulated. This harmonized version attempts to reconcile differences between the two versions and incorporates input and feedback from both translators, disease-specific specialists, and other members of the research team. In our study, the study team, comprised of researchers, nurses, and community advisory board members reviewed the concepts related to health-related quality of life, and then discussed each instrument question-by-question to ensure that consensus was reached on the cultural validity and fidelity of the translated version.

Issues that came up during the translation centered on variations of concepts (such as emotions) that were difficult to translate, or activities that are common in the West but not relevant to life in Thailand. For example, in the MOS-HIV, 'down in the dumps' was translated to mean 'really depressed.' Translators also had difficulty with some English idioms such as 'weighed down by health problems,' which was originally inappropriately translated to mean 'losing weight due to health problems'. The final version used a Thai translation to 'be burdened by health'. In the SF-12, it was necessary to emphasize the words 'physical' and 'mental' in questions such as 'accomplished less because of your physical health' or 'accomplished less because of mental state' to clarify the separate domains of health being asked about. Finally, both questionnaires had difficulty with the English concept 'peaceful' or 'calm', which was ultimately translated as being 'stable, feel OK' ("nim" in Thai).

Regarding activities present in both questionnaires Thai translators and research team members noted that climbing several flights of stairs, bowling, and walking one block were not relevant in Thai society. Most Thai cities or villages do not have standard city blocks and some villages do not have buildings with many stairs, so it is difficult for Thai people to quantify what one flight of stairs is. Also, bowling is an uncommon past time. Instead of being translated literally, the translation and research team agreed upon equivalent activities. For example, 'walking one block' became 'walking from one electric pole to the next' and 'climbing several flights of stairs' became 'walking up a hill.'

The final instruments were agreed upon by all parties before being pilot tested among HIV+ patients at outpatient clinics in Northern Thailand.

### Subjects

The target sample for the pilot study was 100 HIV+ patients receiving ARV at two local HIV outpatient hospital clinics in northern Thailand.

Interviewers approached consecutive patients after they had completed their appointments with health care personnel and invited them to participate. If they agreed, the interviewers attempted to obtain informed consent. Inclusion criteria for participation in the study consisted of: age 20 years or older, current or past use of HAART, ability to communicate in Thai, and ability to provide consent. Non-Thai speaking patients and patients unable to complete an interview due to impaired mental status were excluded. Five patients declined to participate in the study due to lack of time, transportation issues, or lack of interest in the study.

### Demographic and HIV/AIDS related information

Demographic information, including age, sex, marital status, educational attainment, and occupation/employment status were collected from each patient. Patients were also asked to report the date and results of their last CD4 cell count. In addition, each patient was asked to estimate the number of days in the past three months that illness had kept him or her in bed (bed days). A second question asked about the number of days in the past three months that the patient was required to reduce his or her daily activities due to illness.

### Procedures

Each patient completed either the MOS-HIV or the SF-12. All patients completed the symptom questionnaire. A mediated self-administration technique was suggested and used to administer the interviews. Interviewers sat side by side with the patients and read questions out loud to the patient while the patient read along with the interviewer using his or her own copy of the questionnaire. The patient then filled in his or her response on his or her personal copy of the questionnaire.

### Statistical Analysis

Chi-square and T-tests were used to compare characteristics of patients in the two samples. Scale distributions for each scale score within the MOS-HIV and SF-12 and the proportion of minimum and maximum responses were determined to assess the impact of floor and ceiling effects for each scale.

The reliability, or scale internal consistency, of both questionnaires was evaluated by calculating Cronbach's alpha for the multi-item scales. In our study, a Cronbach's alpha of 0.7 or greater was considered acceptable for group comparisons. Known groups validity testing was also conducted for the summary scores of the SF-12 and MOS-HIV using a series of dichotomized variables for CD4 count, SDM score, number of bed days, and number of days of reduced activity. CD4 count was dichotomized at the approximate median (200 cells/mm3); symptom score was also dichotomized at the median (13.0). Bed days and number of days of reduced activity were dichotomized as zero days vs. any (one or more) days. We hypothesized that the mean summary scores for both the SF-12 and MOS-HIV would be weakly to moderately correlated with each variable.

## Results

### Participant Characteristics

#### Overall

Of the 100 pilot test participants, the majority were female (58.0%), married (53.0%), employed as laborers (57.3%), and had completed only primary education (60.0%). The participants' median age was 36 years, as shown in Table 1.

**Table 1:** Selected Participant Characteristics by MOS-HIV and SF12 Questionnaire

		QOL tool				Group Total	
				
Characteristics							
		MOS-HIV (n = 50)		SF12 (n = 50)		(N = 100)	
		Count	Col %	Count	Col %	Count	Col %
Sex	Male	17	34.0%	25	50.0%	42	42.0%

	Female	33	66.0%	25	50.0%	58	58.0%

Marital status	Single	2	4.0%	8	16.0%	10	10.0%

	Married*	22	44.0%	31	62.0%	53	53.0%

	Widowed*	24	48.0%	9	18.0%	33	33.0%

	Divorced	2	4.0%	2	4.0%	4	4.0%

Education	Primary education	30	60.0%	30	60.0%	60	60.0%

	Middle School	6	12.0%	7	14.0%	13	13.0%

	High School	9	18.0%	6	12.0%	15	15.0%

	Vocational Ed	4	8.0%	5	10.0%	9	9.0%

	Bachelor degree	1	2.0%	2	4.0%	3	3.0%

Occupation	Manual Labor	26	52.0%	23	46.0%	49	49.0%

	Agriculture	3	6.0%	5	10.0%	8	8.0%

	Office work	2	4.0%	3	6.0%	5	5.0%

	Self employed	5	10.0%	10	20.0%	15	15.0%

	Government employee & State Enterprise	1	2.0%	2	4.0%	3	3.0%

	House work	4	8.0%	3	6.0%	7	7.0%

	Unemployed	9	18.0%	4	8.0%	13	13.0%

ART Taken	No			2	4.0%	2	2.0%

	Yes	50	100.0%	48	96.0%	98	98.0%

Age	Median	35.5		36		36	

	Mean ± SD	36.3 ± 5.7		37.9 ± 8.1		37.1 ± 7.0	

	Min - Max	26 - 51		26 - 64		26 - 64	

		(n = 40)		(n = 42)		(N = 82)	
CD4 cell count	0 - 100	11	27.5%	8	19.0%	19	23.2%

	101 - 200	14	35.0%	13	31.0%	27	32.9%

	201 - 300	7	17.5%	10	23.8%	17	20.7%

	301 - 500	7	17.5%	7	16.7%	14	17.1%

	> 500	1	2.5%	4	9.5%	5	6.1%

	Median	142.0		209.0		174.5	

	Mean ± SD	189.25 ± 138.80		246.00 ± 177.50		218.32 ± 161.36	

	Min - Max	0 -550		17 - 700		0 - 700	

Signs and Symptoms Score (0 - 88)	Median	15.0		10.50		13.0	

	Mean ± SD	16.70 ± 11.17		13.72 ± 10.47		15.21 ± 10.88	

	Min - Max	1 - 61		0 - 51		0 - 61	

Number of days spent in bed in past 3 months	None	38	76.0%	40	80.0%	78	78.0%

	1 - 2 days	6	12.0%	5	10.0%	11	11.0%
	> 2 days	6	12.0%	5	10.0	11	11.0%
Number of days of reduced activity due to illness in past 3 months	None	40	80.0%	39	78.0%	79	79.0%

	1 - 2 days	1	2.0%	4	8.0%	5	5.0%

	> 2 days	9	18.0%	7	14.0%	16	16.0%

*chi-square, p < 0.05

The mean self-reported CD4 cell count was 218.2 ± 161.4 cells/mm^3^, with a median of 2.9 months since the most recent CD4 test. The majority of patients (76.8%) reported CD4 counts less than or equal to 300, with the greatest number of participants (32.9%) reporting counts between 101-200.

#### Patient Characteristics by questionnaire module

Table 1 provides participant characteristics stratified by quality of life battery completed. There were significantly more married patients in the SF-12 group and widowed patients in the MOS-HIV group. Otherwise there were no statistically significant differences between the groups. Participants completing the MOS-HIV had a median age of 35.5 years, compared to the median age of 36.0 years for those completing the SF-12. Those who completed the MOS-HIV were predominately female (66%). SF-12 respondents were divided equally between males and females. Proportionately, respondents to each questionnaire had approximately the same educational attainment with 60.0% of those completing each QOL battery having finished at least primary education. The majority of individuals - 58.0% of those completing the MOS-HIV and 56.0% of those completing the SF-12- earned their livelihoods through farming or manual labor. Just under half (48%) of those completing the MOS-HIV were widowed and 44% were married. The majority (62%) of those completing the SF-12 were married, with 18% being widowed.

The mean self-reported CD4 cell count was 189.3 ± 138.8 cells/mm^3 ^for respondents to the MOS-HIV and 246.0 ± 177.5 cells/mm^3 ^for respondents to the SF-12.

### Psychometric Properties

The median times to complete the quality of life components alone were 11.0 minutes to complete the MOS-HIV and 5.0 minutes to complete the SF-12. The response rate for both the MOS-HIV and SF-12 was 100%, with no missing answers for any of the questions. The symptom score questionnaire also had a very high response rate, with only 1% missing data for each item. Compared to the MOS-HIV and SF-12, there was a more skewed distribution towards the lower end of the scale, with a majority of participants responding, "No, I do not experience this symptom."

### Scale Distributions

The mean and median summary and subscale scores of each of the QoL questionnaires are given in Table 2.

**Table 2:** Scale Descriptive Data

Subscale	Number of items	Mean	Median	SD	α	25%	75%	% Floor	% Ceiling
**MOS-HIV**									
									
Physical Health Summary Score (PHS)	n/a	53.1	54.6	7.2	0.54	50.28	58.91	0	0
									
Mental Health Summary Score (MHS	n/a	53.4	54.9	7	0.51	48.31	58.01	0	0
									
General Health	5	56.8	60	15.3	0.46	48.75	65	0	0
									
Pain	2	74.2	77.8	20.5	0.6	55.56	100	0	26
									
Physical Function	6	88.7	91.7	17.2	0.78	83.33	100	0	48
									
Role Function	2	88	100	21.6	NC	87.5	100	0	76
									
Social Function	1	84.8	100	27.6	NC	80	100	2	70
									
Mental Health	5	74.9	76	15.7	0.67	64	84	0	10
									
Energy & Fatigue (Vitality)	4	73.2	75	16.2	0.63	63.75	85	0	6
									
Health Distress	4	82.4	87.5	17.7	0.83	73.75	100	0	26
									
Cognitive Function	4	78.7	80	16.3	0.65	68.75	91.25	0	12
									
Quality of Life	1	80	75	16.8	***	75	100	0	30
									
Health Transition	1	72	75	24.6	***	50	100	0	38
									

**SF-12**									
									
Physical Health Summary Score (PCS)	n/a	49.03	49.58	7.5	***	44.78	54.93	0	0
									
Mental Health Summary Score (MCS)	n/a	45.63	46.28	9.1	***	39.01	51.65	0	0

#### MOS-HIV

The mean physical health summary score (PHS) was 53.1 and the mental health summary score (MHS) was 53.4. Very limited floor effects were found in the MOS-HIV, with 2.0% of the participants providing the lowest possible score for the social functioning (SF) subscale. Ceiling effects were more pronounced. The only subscale that had 0% of respondents providing the highest possible score was the general health perceptions (GHP) subscale. The remaining ten scales ranged from 6.0% (energy/fatigue, vitality [EF]) to 76.0% (role functioning [RF]) of the respondents reporting the highest possible score of 100.

#### SF-12

The mean PCS was 49 and the mean MCS was 45.6. There were no floor or ceiling effects found for the SF-12 summary score during this pilot test.

### Evidence for Reliability

The internal consistency coefficients for the multi-item scales are shown in Table 3. Cronbach's alpha coefficients were greater than 0.7 for all of the scales of the MOS-HIV, except for the physical functioning (PF) subscale, which approached acceptability at 0.67. Since subscale scores are not generated for the SF-12, alpha coefficients were not estimated.

**Table 3:** Inter-correlation among mean scale scores and Cronbach's alpha coefficients, MOS-HIV

Scale	Mean score	PHS	MHS	PF	SF	RF	CF	P	MH	EF	HD	QL	GHP	HT
PHS	53.1	**0.76**												
MHS	53.4	0.56	**0.76**											
PF	88.7	0.83	0.48	**0.67**										
SF	84.8	0.69	0.47	0.39	**0.77**									
RF	88.0	0.72	0.41	0.70	0.37	**0.76**								
CF	78.7	0.42	0.48	0.37	0.32	0.25	**0.77**							
P	74.2	0.53	0.35	0.31	0.11	0.13	0.27	**0.78**						
MH	74.9	0.24	0.79	0.25	0.19	0.21	0.12	0.32	**0.77**					
EF	73.2	0.62	0.79	0.61	0.31	0.44	0.33	0.37	0.52	**0.75**				
HD	82.4	0.29	0.81	0.18	0.28	0.18	0.35	0.40	0.65	0.52	**0.76**			
QL	80.0	0.55	0.67	0.59	0.33	0.45	0.20	0.15	0.33	0.67	0.32	**---**		
GHP	56.8	0.60	0.53	0.33	0.34	0.41	0.08	0.43	0.40	0.45	0.34	0.40	**0.76**	
HT	72.0	0.27	0.17	0.27	0.28	0.17	0.10	-0.12	-0.11	0.15	-0.01	0.44	0.23	**---**

### Evidence for validity

Table 4 shows correlations of the MOS-HIV and SF-12 questionnaire subscale scores with patient-reported CD4, symptom score, number of days spent in bed, and number of reduced activity. As hypothesized, symptom score, number of days spent in bed, and number of days of reduced activity were all negatively correlated with subscale scores. This provides evidence for the construct validity of the subscales as a measure of health status.

**Table 4:** Scale score correlations with patient reported clinical markers

	CD4	Symptom score	Days spent in bed	Days of reduced activity
**MOS-HIV**				

PHS	0.18	-0.58	-0.68	-0.41

MHS	0.25	-0.63	-0.41	-0.18

GHP	0.19	-0.43	-0.13	-0.18

BP	0.55	-0.51	-0.44	-0.43

PF	0.30	-0.48	-0.74	-0.46

RF	0.26	-0.44	-0.53	-0.26

SF	-0.04	-0.39	-0.40	-0.09

MH	0.25	-0.51	-0.26	-0.23

EF	0.32	-0.41	-0.48	-0.29

HD	0.07	-0.53	-0.32	-0.06

CF	0.14	-0.49	-0.36	-0.05

QL	0.22	-0.37	-0.31	-0.19

HT	-0.03	-0.14	-0.12	0.16

**SF-12**				

PCS	0.11	-0.39	-0.36	-0.38

MCS	-0.05	-0.55	-0.57	-0.10

Known groups validity testing was done to compare the SF-12 and MOS-HIV physical and mental health summary scores to variables measuring health status. These variables included CD4 count, symptom score, number of days spent in bed, and number of days of reduced activity. F-scores were calculated using ANOVA of differences between each scale and each variable.

For CD4 group, neither the MOS-HIV or SF-12 achieved significance for discrimination between the groups. The p-values for the MOS-HIV were slightly smaller than those for the SF-12 (MOS-HIV PHS = 0.29 vs. SF-12 PCS = 0.51; MOS-HIV MHS p = 0.20 vs. SF-12 MCS p = 0.84).

Both the MOS-HIV and SF-12 scales were successful at discriminating between these groups with all summary scores' reaching significance (MOS-HIV PHS p = 0.002, MOS-HIV MHS p = 0.001, SF-12 PCS p =.015, and SF-12 MCS p = 0.00)

Only the PHS score discriminate number of days of reduced activity (0 days vs. any days, p = 0). P-values for other comparisons were PCS (p = 0.082), MHS (p = 0.097), and MCS (0.633).

Both the MOS-HIV and SF-12 physical health summary scores were able to discriminate groups defined by number of days spent in bed in the past three months (0 days vs. any days). The p-values for the PHS and PCS were 0.001 and 0.003 respectively: MHS and MCS were not significant (0.092 and 0.104 respectively).

## Discussion

The results of this initial test provide evidence for acceptable reliability and validity of the Thai versions of the MOS-HIV and SF-12 as measures of HRQOL among HIV+ patients in Northern Thailand. Both questionnaires were successfully translated/culturally adapted into the Thai language. The low refusal rate (4%) and high response rate for all questionnaires (99-100%) point to good acceptability by patients.

In this study, trained interviewers adopted a modified face-to-face technique where the questionnaire was read out-loud to the patient, with the patient then filling in his or her response. This modified technique was well-received by both interviewers and interviewees and allowed interviewees to ask questions or have issues clarified during the interview process. By filling out the answers themselves, however, confidentiality was preserved. Furthermore, in Thai and other Asian cultures, deference to authority (such as the interviewer) and the desire to minimize interpersonal conflict may influence patient response in interviewer-administration as interviewees will try to answer questions in a way they think will be socially acceptable to the interviewer. Self-administering the questionnaires may have allowed interviewees to be more truthful in their answers.

Psychometric performance was consistent with previous studies and suggested that there was adequate internal consistency for the MOS-HIV [12-17]. Evidence for construct validity was demonstrated by the relationship between summary scores and relevant clinical variables, including symptom distress score, number of days spent in bed, and days of reduced activity. Interestingly, CD4 count was the only variable which was not significantly correlated to either physical or mental health summary scores. This is consistent with most previous research studies [17,18] and is unsurprising as many patients with low CD4 on HAART may be asymptomatic, while some of those responding to treatment may have diminished scores due to side effects of HAART therapy. Also as expected, the physical health scores were better able to distinguish between the variables examined, which reflect physical rather than mental functioning.

It was interesting to note that the MOS-HIV performed slightly better than the SF-12 in its ability to discriminate between known groups with a greater number of significant p-values (4 vs. 3) and p-values approaching significance. However, one must be cautious in interpreting these results, given the different samples of patients completing the two questionnaires.

Because we were interested in the ultimate usability of health-related quality of life questionnaires in clinical settings, we selected tools that differed in length. The more comprehensive MOS-HIV provides more precise estimates of a range of specific issues. However, it is more time-consuming than the SF-12 (11 vs 5 minutes on average). Investigators and clinicians should weigh these factors in selecting between the two alternatives.

A study published in 2004 evaluating the psychometric properties of an independently translated version of the MOS-HIV found a high level of internal consistency reliability of multi-item scales with all multi-item scales achieving Cronbach's alpha of 0.7 or above [14]. In the 2004 study, respondents were recruited only from PLWHA support or self-help groups. Our study was conducted in community-based hospital settings and recruited respondents from patients at their regular visits, which may capture a more representative sample of HIV+ people in Northern Thailand. Notably, many patients had very low (<300) CD4 counts. However, the overwhelming majority (98%) of patients participating in our study were receiving antiretroviral therapy, which was rare during the time of the study. At the time of this study, there was no translated version of the SF-12 available. However, there is now an official translation of this instrument. Future studies should be conducted to confirm the performance of the SF-12 Thai version.

Limitations of our study included an inability to test for responsiveness, as all questionnaires were tested among patients at a single time point. Since patients completed either the MOS-HIV or the SF-12 and not both, we were unable to perform head-to-head comparisons of the two instruments or correlate them to each other. Although our study indicated that the MOS-HIV performed slightly better than the SF-12 in its ability to discriminate between known groups, this could be a random effect since different groups filled out different questionnaires. In addition, because we did not have access to patient medical records or providers, we asked patients to self-report their CD4 count. There was individual variability in the extent to which patients could report these. However, we only used this variable to test the construct validity of the quality of life scales. Nonetheless, it is likely to have introduced additional random error into the estimate of CD4 count and attenuated the correlation with quality of life scores.

There is often little time in clinic settings for health care personnel to administer questionnaires. Outside of clinical trials, there is little attention paid to measurements of HRQOL. However, more health care workers are recognizing that HRQOL can play a large impact on HIV treatment and care, especially in the area of compliance with complicated and potentially toxic drug regimens. As both questionnaires were well-accepted by patients in this pilot test, in our own ongoing studies, we are using the MOS-HIV at yearly intervals or at certain milestones in a patient's care, such as immediately before commencing antiretroviral therapy. At other visits, a shorter tool, such as the SF-12 can provide a snapshot of the patient's QOL. By focusing a part of the clinical encounter on QOL, the health care team is given an opportunity to address issues that may not be captured during a standard HIV clinic visit. Most importantly, the patient is provided a dedicated outlet to bring attention to concerns that may affect his or her care with the team.

In conclusion, the Thai versions of the SF-12 and MOS-HIV were acceptable, valid, and reliable among HIV+ Thai patients interviewed in a clinical outpatient setting. As more Thais are able to access ARV through national health care programs designed to cover treatment for PLWHA's, attention to QOL will become increasingly important to provide effective and patient-centered care. The SF-12 and MOS-HIV are promising tools that may be used in clinical environments to help assess patient's HRQOL and guide both patients' and providers' decision-making.

## Competing interests

The authors declare that they have no competing interests.

## Authors' contributions

SC obtained funding, oversaw the parent study, and assisted in writing the manuscript. TW drafted the manuscript and assisted in data analysis. SK was responsible for the overall data management and study procedures. CR assisted with translation and cultural adaptation. VK assembled the measurement battery and produced study materials. AW obtained funding for the substudy, oversaw study design and analysis, and revised the manuscript. All authors read and approved the final manuscript.
